# Benchmarking network algorithms for contextualizing genes of interest

**DOI:** 10.1371/journal.pcbi.1007403

**Published:** 2019-12-20

**Authors:** Abby Hill, Scott Gleim, Florian Kiefer, Frederic Sigoillot, Joseph Loureiro, Jeremy Jenkins, Melody K. Morris

**Affiliations:** 1 Chemical Biology and Therapeutics, Novartis Institutes for Biomedical Research, Cambridge, Massachusetts, United States of America; 2 Novartis Informatics, Novartis Institutes for Biomedical Research, Basel, Switzerland; 3 Respiratory Disease Area Department, Novartis Institutes for Biomedical Research, Cambridge, Massachusetts, United States of America; Chinese Academy of Sciences, CHINA

## Abstract

Computational approaches have shown promise in contextualizing genes of interest with known molecular interactions. In this work, we evaluate seventeen previously published algorithms based on characteristics of their output and their performance in three tasks: cross validation, prediction of drug targets, and behavior with random input. Our work highlights strengths and weaknesses of each algorithm and results in a recommendation of algorithms best suited for performing different tasks.

## Author summary

In our labs, we aimed to use network algorithms to contextualize hits from functional genomics screens and gene expression studies. In order to understand how to apply these algorithms to our data, we characterized seventeen previously published algorithms based on characteristics of their output and their performance in three tasks: cross validation, prediction of drug targets, and behavior with random input.

This is a *PLOS Computational Biology* Benchmarking paper.

## Introduction

In 2000, Schwikowski et al. demonstrated the utility of the guilt by association principle to assign function of yeast genes by examining the function of neighboring genes in a protein-protein interaction [1]. Since then, the scientific community has launched a massive effort to determine protein-protein interaction (PPI) networks for model organisms [2–5] and humans [4, 6]. At the same time, a multitude of computational approaches have been developed for contextualizing genes of interest with known molecular interactions in order to aide interpretation of high throughput data. The promise of these algorithms is to connect genes of interest into functional networks and extend the findings with additional genes relevant to the initial list.

In our labs, we aimed to use these algorithms to contextualize hits from functional genomics screens. The hits from a functional genomic screen represent a list of genes that affect a given cellular phenotype (eg. survival [7], autophagy [8], etc.) and that are hypothesized to belong to pathways involved in regulating the phenotype. In these screens, false negatives are also a common concern. In the case of false negatives, genes that affect a given phenotype are missing from the final gene list due to technical factors (eg. editing efficiency) or biological factors (eg. gene redundancy). We aimed to use network algorithms in combination with a protein-protein interaction (PPI) network to both organize hit lists into pathways and extend the hit list through the identification of potential false negatives (i.e. genes that are connected to hits through many PPIs but missing from the hit list).

While many of these network contextualization algorithms have been developed in academia in the context of specific biological questions [9, 10], others are part of commercially available tools (eg. Metacore, Ingenuity Pathway Analysis). However, despite the growing number of available algorithms, to our knowledge there has been no systematic effort to benchmark their ability to return meaningful, actionable hypotheses. In this work, we evaluate network contextualization algorithms available in the Computational Biology for Drug Discovery (CBDD) R package developed by Clarivate, Inc. While we were initially interested in applying these algorithms to hits from functional genomics screens, we appreciated that these algorithms might have utility for other data types with similar interpretation (eg. genes genetically associated to a disease) or for different tasks altogether (eg. target prediction from gene expression signatures). Thus, we assessed the algorithms for three data types: genetic associations; hits from functional genomics CRISPR screens; and gene expression signatures of drug response. We first characterized the algorithms in terms of the novelty and number of connections (i.e. degree) of returned output nodes. We then assessed their performance using cross validation and target prediction, with the ultimate aim of applying appropriate algorithms to contextualize gene lists from gene expression studies or functional genomics screens.

## Results

### Overview of benchmarking workflow

**Fig 1:**
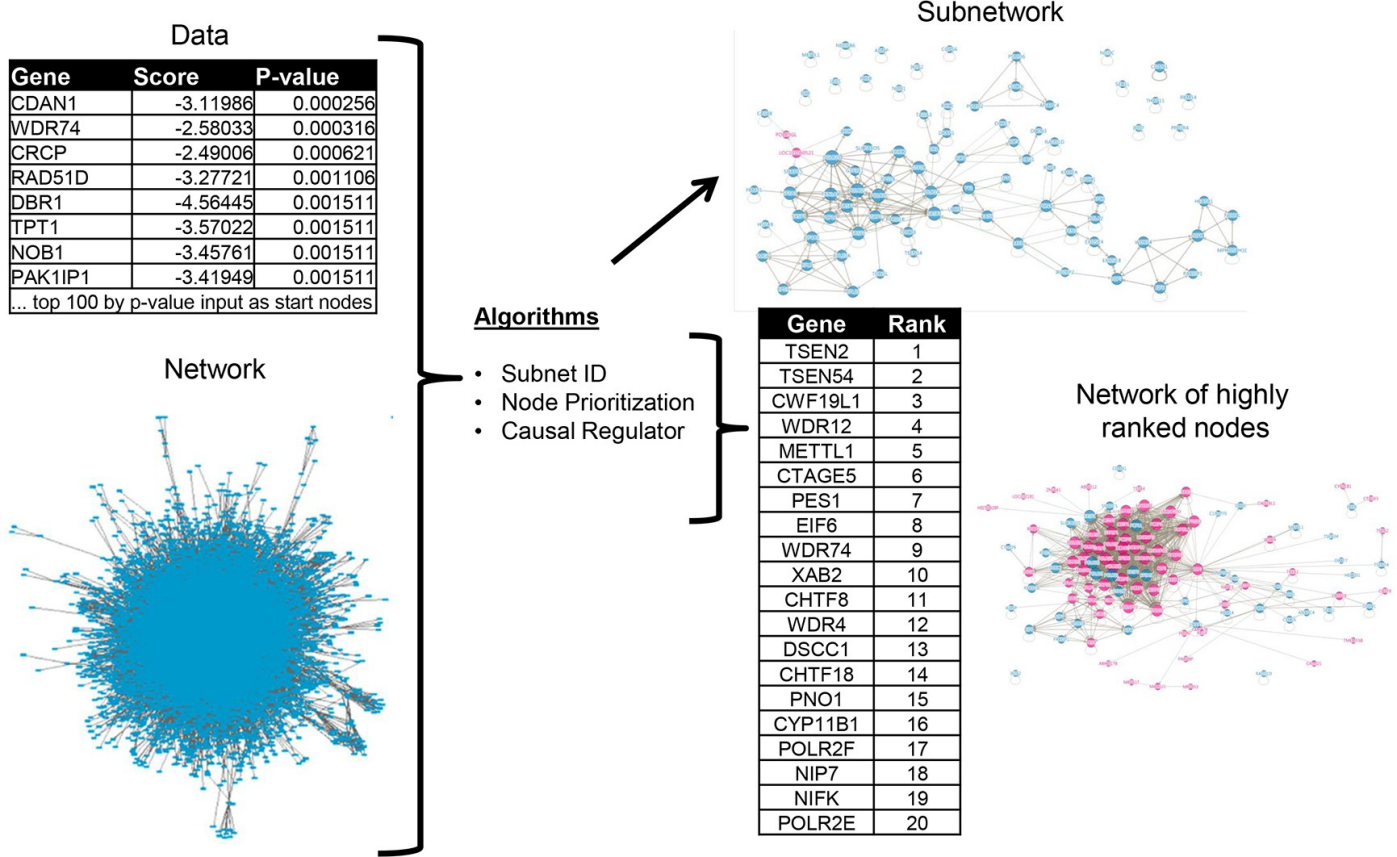
Overview of network algorithm benchmarking workflow: All algorithms considered in this work required a set of identified genes of relevant to a disease, pathway, or treatment (i.e. “start nodes”) as inputs while some also required fold changes and/or p-values. The output of algorithms differed depending on algorithm class, with subnetwork ID algorithms returning highly connected subnetworks; node prioritization algorithms returning ranked lists of genes; and causal regulator algorithms returning ranked lists of hypotheses corresponding to a positive or negative effect of a given gene on the observed data. In the case of node prioritization and causal regulator algorithms, we considered the “output nodes” as the top ranked nodes using a rank cutoff equal to the number of input start nodes for each data set. Also, we note that subnetworks could be constructed from the interactions among the most highly ranked genes in the output lists. For illustration purposes for this figure, we have used the list of top 100 hits (based on p-value) from a CRISPR survival screen in the KBM7 cell line [7]. Each output network contains genes that were included in the input start node list (blue) as well as genes that were identified by the algorithms (pink).

This work evaluates the ability of seventeen algorithms to use a protein-protein interaction (PPI) network to contextualize and extend a list of genes of interest. Fig 1 exemplifies our workflow with a published pooled CRISPR screen of survival [7]. In this case, the hits from the screen were provided to the network algorithms as the input “start nodes”. The type of output depended on the type of algorithm under investigation. In the case of node prioritization and causal regulator algorithms, the output consisted of a list of ranked network nodes (i.e. the “output nodes”) while subnetwork ID algorithms returned a sub-network consisting of output nodes and the connections between them.

In this work, we considered seventeen algorithms (Table 1) implemented as part of the Computational Biology for Drug Discovery (CBDD) collaboration between Clarivate Analytics and sixteen pharmaceutical companies. A key deliverable of CBDD is the CBDD R package which implements published algorithms in a consistent interface. Algorithms chosen were available in CBDD version 8.2 and had no major performance considerations that would limit systematic benchmarking efforts. Additionally, the aim of these algorithms was consistent with our aim: to use the network to contextualize and extend genes of interest.

**Table 1:** Algorithms evaluated.

Algorithm	Category	Network Requirment	Brief Description	Reference
***Node Prioritization Algorithms*: *ranks nodes in the network based on connectivity or distance from start nodes***				
Random Walk	Node Prioritization		Models path of a random walker starting from nodes of interest and walking to other nodes based on edges in the network	[16]
Network Propagation	Node Prioritization		Random walk based approach controlled for degree of nodes	[17]
ToppNet KM	Node Prioritization	Directed	Random walk-based method with limited number of steps	[18]
ToppNet HITS	Node Prioritization	Directed	Random walk-based method that also takes into account hubness and authority of nodes	[18]
Overconnectivity	Node Prioritization		Enrichment of start nodes and gene sets consisting of each network nodes’ neighbors	N/A
Interconnectivity	Node Prioritization		Enrichment based method that identifies nodes between other nodes	[19]
Hidden Nodes	Node Prioritization		Enrichment based method that uses shortest paths to identify nodes between other nodes	[20]
GeneMania	Node Prioritization		Ranks nodes by topological closeness to start nodes in an integrated network	[21]
Guilt By Association	Node Prioritization		Fraction of neighbor nodes that appear in the start node list	[1]
Neighborhood Scoring	Node Prioritization		Guilt-by-association based approach with optional weighting for start nodes	[22]
***Causal regulator algorithms*: *ranks nodes based on evidence that a perturbation to the node would result in observed changes in start nodes***				
Causal Reasoning	Causal Regulator	Signed and Directed	Processes network and calculates directional consistency and overconnectivity with start nodes	[23, 24]
SigNet	Causal Regulator	Signed and Directed	Processes network and calculates several metrics to infer relationship with start nodes	[25]
***Subnetwork ID algorithms*: *extract a part of the input network containing many start nodes and additional connecting nodes***				
DIAMOnD	Subnetwork ID		Evaluates overconnectivity enrichment iteratively until it reaches a user-defined number of nodes	[26]
Pathway Inference	Subnetwork ID		Heuristic methods that identifies subnetworks enriched in start nodes	[27]
Active Modules	Subnetwork ID		Memetic algorithm with addition of encoding/decoding scheme and local search operator	[28]
CASNet	Subnetwork ID	Signed	Considers edge sign to determine relevance to provided start nodes	[29]
HotNet1	Subnetwork ID		Diffusion based method accounting for FDR	[30]
HotNet2	Subnetwork ID	Directed	Extension of HotNet1 approach than incorporates insulated diffusion and edge direction	[31]
Start Node Links	Subnetwork ID		Directly extracts connections between start nodes	N/A

When considering these algorithms, we noted they could be divided into three main categories: (1) node prioritization algorithms that prioritize network nodes that are near input nodes, where the definition of "near" varies depending on the specific algorithm, (2) causal regulator algorithms that prioritize network nodes that regulate input start nodes based on their network connectivity, and (3) subnet identification (ID) algorithms that identify regions of the network that connect input nodes and include additional nodes for their connection if warranted. In the case of subnetwork identification algorithms, we wanted to be able to compare to the simplest case of network connections between nodes. Thus, we include output from an algorithm called “Start Node Links”, which connects input start nodes to each out.

We applied the algorithms to hundreds of datasets from four sources, aiming to test the algorithms on a large selection of data sets of different types and confidences. Initial characterization was performed using three types of data meant to capture phenotype- or disease-relevant pathways: (1) KEGG and REACTOME pathway genesets provide high-confidence, well characterized data sets; (2) DisGeNET provides data sets describing curated disease-gene associations [11, 12]; and (3) hits from phenotypic CRISPR screens provide a source of real experimental data most similar to our intended use case. We then turned our attention to Connectivity Map gene expression response signatures, where the aim of applying the algorithms is to predict the target of a perturbation from the response signature. The network used in this work was a protein-protein interaction network derived by combining multiple sources: the STRING [13, 14] public database, the Metabase (Clarivate) manually curated database, and interactions from affinity purification mass spectrometry experiments (Bioplex [15]).

### Algorithms differ in ranking of start nodes

**Fig 2:**
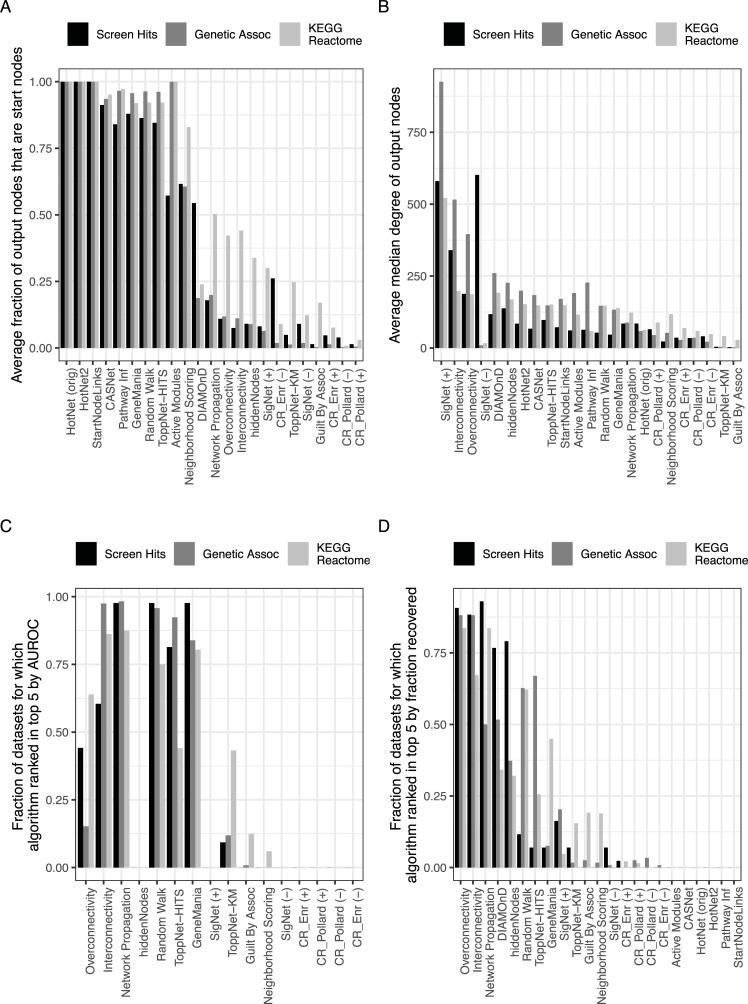
Characterizing algorithms using average fraction of start nodes in the output to indicate tendency to return start nodes in output (A, top left) and degree to indicate tendency to return nodes with many edges (B, top right). Cross-validation performance of algorithms as indicated by the fraction of datasets for which the algorithm appeared in the top five when ranked by AUROC (C, bottom left) or Fraction recovered (D, bottom right). For the fraction recovered analysis, the top nodes were defined as the 200 top-ranked nodes for node prioritization and causal regulator algorithms or any node present in a subnetwork for subnetwork ID algorithms.

To determine which algorithms extended the list of interesting genes beyond the input list provided, we first sought to determine the proportion of output nodes that were contained in the input start nodes (Fig 2A). Within the node prioritization algorithms, Random Walk, ToppNet HITS, and GeneMANIA showed clear tendency to include start nodes in their outputs. While Neighborhood Scoring showed an intermediate behavior, all other node prioritization algorithms did not rank start nodes highly and, rather, tended to include a large number of non-start nodes in their output. As causal regulator algorithms are intended to identify nodes that influence the start nodes, possibly from several steps away, they generally did not have a strong preference for including the start nodes themselves in output lists. Most subnetwork ID algorithms showed a strong tendency to include start nodes in their output with the exception of DIAMOnD, which employs the overconnectivity node prioritization algorithm iteratively until it reaches a user-defined number of nodes (in this case 200).

### Algorithms differ in preference for node degree

We also sought to understand which algorithms had a tendency to include high degree nodes in the output (i.e. “hub nodes”). Hub nodes are those with many edges (or connections) to other nodes. Across all algorithms, several returned extremely high-degree outputs: DIAMOnD, Interconnectivity, and Overconnectivity (Fig 2B). We noted that these algorithms with high-degree outputs are all enrichment-based methods. Other subnetwork ID and node prioritization algorithms had intermediate but rather variable median degree within the outputs. Several of these algorithms (eg. Pathway Inference, CASNet, HotNet, HotNet2, Active Modules, and GeneMANIA) also ranked start nodes very highly, so the median degree of the output depended heavily on the degree of the start nodes. Of the remaining algorithms that showed intermediate behavior by this metric (ToppNet HITS, Hidden Nodes, Random Walk, and Network Propagation), all are walk-based.

### Assessing algorithm performance by cross-validation

In assessment of performance, we performed 10 repeats of 10-fold cross-validation to determine how well the algorithms were able to recover nodes randomly excluded from the input lists. The excluded nodes were true positives in that they were related to the remaining input nodes on the basis of their membership in the original list. Thus, this test determined the ability of the algorithms to identify nodes biologically related to the input list. To summarize the results from cross validation, the area under the receiver operator curve (AUROC) is often evaluated. This metric assumes a perfect gold standard and takes into account both the true positives with the sensitivity metric and false positives with the specificity metric. However, we noted that our input lists were not perfect gold standards in that some nodes returned by the algorithms might appear to be false positives but actually be biologically related to the input list (i.e. nodes designated as false positives by the specificity calculation might actually be false negatives in the original input list). Thus, we also computed the fraction of excluded nodes that were recovered in the top 200 nodes returned by each algorithm (i.e. the fraction recovered). This metric does not take into account false positives and instead asks the question relevant to our intended use of the algorithm: if we were to follow up on the top 200 nodes returned by the algorithms, would nodes known to be biologically relevant to the initial input list be recovered? It is equivalent to the true positive rate (i.e. sensitivity) computed when the top 200 nodes returned by the algorithm are considered the output of the algorithm.

We calculated the AUROC and fraction recovered for each data sets tested. To summarize across individual data sets, we noted that variability in the metrics across datasets made it difficult to determine which were performing better than others (S1 Fig). Thus, we used a ranked-based approach and found the fraction of data sets for which each algorithm appeared in the top five when ranked by AUROC or fraction recovered (Fig 2C and 2D). While performance by AUROC varied across data sources, Random Walk, Network Propagation, GeneMANIA, Interconnectivity, and ToppNet HITS performed among the top node prioritization algorithms in all datasets tested. Subnetwork ID algorithms could only be quantified by fraction recovered, and for these algorithms, a node was considered ‘recovered’ if it was returned in any subnetwork (in contrast to node prioritization outputs, which were limited to the top 200 nodes). While several different algorithms performed better by the fraction recovered metric than AUC (*eg* Overconnectivity and Hidden Nodes), the walk-based algorithms Network Propagation and Random Walk performed well by both metrics in all datatypes considered here.

### Behavior of algorithms with random input lists

In order to determine whether certain nodes, particularly hub nodes, would be highly ranked by a given algorithm regardless of the input list, we ran the algorithms on 10,000 randomly selected input start node lists. We then compiled the output and calculated the fraction of times that each node appeared in most highly ranked nodes. For most algorithms, a few hundred nodes were ranked in the top 200 nodes in more than 5% of randomly generated list (Table 2). Of greater concern, some algorithms highly ranked a few specific nodes in more than 50% of the output from random input lists (eg. Causal Reasoning, InterConnectivity, SigNet, Random Walk, and ToppNet—HITs), indicating that these nodes were likely to be included in the algorithms’ outputs regardless of their importance for the particular pathway or process of interest. For most algorithms, the tendency of nodes to be highly ranked in the output even with randomly chosen input nodes was related to the degree of the nodes (S2 Fig). However, degree of the nodes did not explain the behavior of all randomly included nodes for all algorithms, and it was clear that other network properties play a role in this finding.

**Table 2:** Number of nodes ranked in top 200 when algorithms were run with 200 randomly chosen nodes as input start nodes.

Algorithm	Number of nodes highly ranked in 50% of random input tests	Number of nodes highly ranked in 5% of random input tests
Causal Reasoning (Pollard Rank)	64	1129
InterConnectivity	44	1042
Hidden Nodes	0	559
SigNet	200	375
Network Propagation	0	309
ToppNet–HITs	239	289
Random Walk	4	200
Guilt by Association	0	119
ToppNet–KM	0	56
Causal Reasoning (Enrichment Rank)	0	0
Overconnectivity	0	0
Neighborhood Scoring	0	0
GeneMania	0	0

### Use of algorithms for target identification using connectivity map

**Fig 3:**
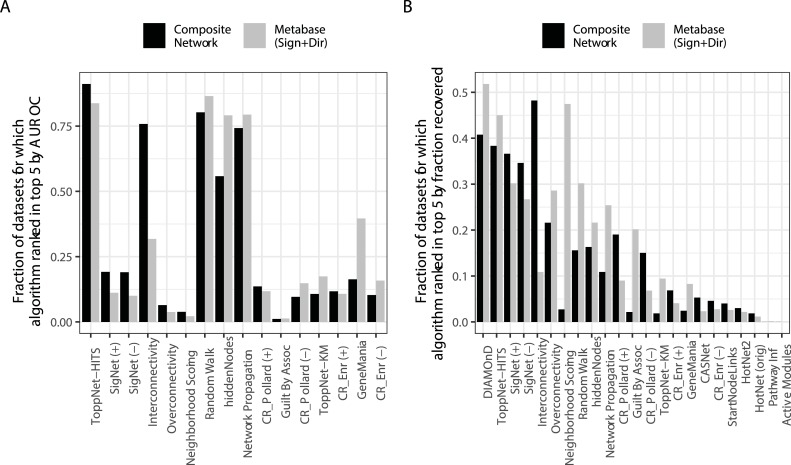
Connectivity Map target prediction in the composite network or metabase signed+directed. Performance was characterized by the ability of the algorithms to highly rank known targets of drugs. (A, top left) Fraction of datasets for which the algorithm appeared in the top five when ranked by fraction of drug targets recovered (B, top right) Fraction of datasets for which the algorithm appeared in the top five when ranked by AUROC.

Because causal regulator algorithms were developed to identify upstream regulators of differentially expressed genes, we tested their ability to accomplish this goal using the Connectivity Map [32]. The Connectivity Map dataset captures gene differential expression after treatment with a drug. Thus, for this analysis, the input start nodes were the differentially expressed genes, and the gold standard we tested was the ability to of the algorithms to highly rank the real target(s) of the drugs used for each treatment condition. Our results (Fig 3) indicated that for this type of data, SigNet appeared in the top ranked algorithms. However, it is important to note that, in general, the causal regulator algorithms did not outperform several node prioritization algorithms. We hypothesized that the causal regulator algorithms relied heavily on network information that was not known with sufficient accuracy in the network, which was a composite of signed, unsigned, directed, and undirected edges from multiple sources. Thus, we ran the connectivity map benchmarking workflow with a network that only contained high confidence, signed, and directed edges from the curated Metabase network. With this network, our conclusions were generally consistent (Fig 3, grey bars) although neighborhood scoring performed much better with the Metabase network than composite network.

## Discussion

Taken together, our results clearly demonstrate the strengths and weaknesses of several algorithms (Table 3). The benchmarking results shown here suggest that certain categories of algorithms may have different applications, and the choice of algorithm(s) may depend on the specific use case. If the scientist is interested in re-ranking or contextualizing input start nodes, Random Walk, GeneMANIA, or subnetwork ID methods perform well. Alternatively, if the scientist aims to extend an input list to identify new nodes that may be involved in a disease process or response, Network Propagation or Overconnectivity would be better selections. Of the causal regulator algorithms, SigNet performed well using one metric for tests of target prediction using connectivity map response signatures. However, we note that several node prioritization algorithms also performed well at this task.

**Table 3:** Summary of Algorithm Characteristics and Performance. “Tunable” indicates that the algorithm contains an tunable parameter directly related to the evaluated aspect. Bold italics are used to indicate algorithms that perform well for the indicated metric with flanking asterisks distinguishing the top performers.

Algorithm	Highly ranks start nodes	Output Degree	Highly ranks nodes with random inputs (number of nodes in 50%/5% of test cases)	Number of datatypes for which algorithm is top for gene list extension (AUROC, FR)	Number of networks for which algorithm is top for target prediction task (AUROC, FR)
Network Propagation	tunable		***0*, *309***	* 3, 2 *	* 2, 0 *
Random Walk	Y, tunable		***0*, *200***	* 3, 2 *	* 2, 0 *
GeneMania	Y		* 0, 0 *	***3*, *1***	***1*, *0***
Interconnectivity		High	44, 1042	* 3, 3 *	***1*, *1***
ToppNet–HITS	Y, tunable		239, 289	***3*, *1***	* 2, 2 *
Overconnectivity		High	* 0, 0 *	* 2, 3 *	***0*, *1***
DIAMOnD	tunable		n/a	***n/a*, *2***	* n/a, 2 *
ToppNet–KM	tunable	Low	***0*, *56***	***1*, *0***	0, 0
Hidden Nodes			***0*, *559***	***0*, *1***	* 2, 1 *
Guilt By Association		Low	***0*, *119***	0, 0	n/a, 0
Neighborhood Scoring	Y, tunable	Low	* 0, 0 *	0, 0	***0*, *1***
Pathway Inference	Y, tunable		n/a	n/a, 0	n/a, 0
Active Modules	Y, tunable	tunable	n/a	n/a, 0	n/a, 0
CASNet	Y		n/a	n/a, 0	n/a, 0
HotNet1	Y, tunable		n/a	n/a, 0	n/a, 0
HotNet2	Y, tunable		n/a	n/a, 0	n/a, 0
Start Node Links	Y		n/a	n/a, 0	n/a, 0
Causal Reasoning		Low	64, 1129 (Pollard)	0, 0	n/a, 0
SigNet		High	200, 375	0, 0	* 0, 2 *

In this work, we have characterized the algorithms’ performance using a wide range of data sources in order to understand the broad behavior of the algorithms. However, it is possible that a specific dataset of interest will require a different algorithm than that recommended by these results. For this work, we limited ourselves to algorithms implemented as part of the CBDD collaboration, since the consistent interface resulting from this effort facilitated well our benchmarking study. However, we note that many additional network algorithms are have been developed in the literature (eg. [33–36]), and a comparison of additional algorithms to those studied here in a future benchmarking effort might further refine our understanding in what type of algorithms are appropriate for various tasks.

The majority of these results were obtained using a large network containing PPIs from multiple sources. However, we note that we have run these same characterizations with multiple networks [37] and have included results from a published, undirected network (HumanNet [38]) for the task of extending an initial gene list to include additional biologically relevant nodes (S3 Fig). The results for the HumanNet analysis are consistent overall with our previous results and indicate that network propagation and random walk are top performing algorithms even with an un-directed network. Our goal with this work was to understand which algorithms performed well for each data type and task. However, another key component to the success of our analysis is the influence of network quality on performance. While we have not undertaken a systematic evaluation of this question with this work, we look forward to future benchmarking efforts to shed further light into this important aspect as well.

Finally, we did not explore individual algorithm parameters, instead relying on author recommendations. However, we note in Table 3 that some algorithms (eg. Network Propagation and Random Walk) contain a parameter meant to alter the number of start nodes included in the output. While a full exploration of parameter landscape for each individual algorithm is out of scope for this work, we have noted key parameters in S1 Table and would encourage developers of novel algorithms to consider the metrics we have explored here as means to characterize their algorithm across its parameter space and as a starting framework for benchmarking a novel algorithm against existing algorithms.

## Materials and methods

### Network algorithm parameters

For each algorithm, parameters were chosen to moderate the behavior of the algorithms (S1 Table). For example, both random walk and network propagation contain a parameter that sets the probability that the random walk will restart at the start nodes at each step; this parameter was set to 0.5 for both to allow for comparison between the two algorithms. If the value of the parameter that would result in moderate behavior was not obvious, it was set based on author recommendations.

### Data sets

In the KEGG and Reactome data sets, all sets with 20 or more nodes were included, yielding 165 sets from KEGG and 307 from Reactome. We also used curated gene-disease associations from DisGeNet [11, 12] (accessed 7 June 2016). Nodes were included in a disease set if they had at least 2 Pubmed IDs, and disease sets were kept if the number of associated genes was at least 20, yielding 117 disease sets. For these data sets, where fold changes and p-values are not available, nodes were assigned a log_2_ fold change of 1 and p-value of 0.05 to allow input lists to be run with algorithms that require fold change or p-value.

To test the algorithms using real experimental data, 43 pooled CRISPR screens from Novartis were used as an example set of experimental data with relatively low noise. For CRISPR experiments, cells were transfected with a GFP-tagged target protein of interest and Cas9, then exposed to a pooled library of sgRNA. Cells were FACS-sorted into high- and low-GFP populations, and sgRNA count was used to calculate fold changes and RSA p-values for each targeted gene [8]. Genes were included in start lists if the RSA p-value < 1x10^-4^ and for each experiment (which may have included multiple comparisons) the start list with length closest to 150 genes was used. Experiments were excluded from the benchmarking data if the longest start list was <20 genes.

The causal regulator algorithms were originally developed to identify proteins upstream of observed gene expression changes. Since this approach was not specifically relevant to the pathway and screening data described above, we also used data from the Connectivity Map [32], with more appropriate parameters for the causal regulator algorithms. Data from the connectivity map (v1) was downloaded from https://portals.broadinstitute.org/cmap/ and genes were included as start nodes if they were differentially expressed more than 2-fold for the indicated treatment. Because connectivity map includes some compounds in multiple settings, we ran the algorithms on each data set independently and then used the average for summarizing algorithm performance.

### Networks

Three different network sources were used for this work: (1) The “Composite network” consisting of high-confidence, PPI or transcription factor-gene interactions from the Metabase manually curated network, STRING [13, 14] and BioPlex [15]; (2) “MetabaseSD” consisting of signed and directed high confidence interactions from the Metabase curated network; and (3) HumanNet a previously published undirected network [38]. The composite network was constructed by combining edges from the indicated sources. In the case of the Metabase curated network, nodes are occasionally mapped to multiple genes. In these cases, multiple edges were included in the composite network to capture all genes represented by that network node. In the case of STRING, only the “STRING:actions” network edges were considered high confident, PPI interactions and included in the composite network. The resultant composite network consisted of 597,538 unique edges. Of these edges, 22.6% were signed and 36.8% were directed. For algorithms that required direction, any undirected edge was considered in both directions. For those that required sign, a positive sign was assumed for un-signed edges.

### Calculation of start node fraction and median degree

For the purposes of these calculations, “output nodes” were considered to be the top *n* nodes ranked by the algorithm, where *n* was the length of the input start list. To quantify preference for start nodes, we calculated the proportion of output nodes that were represented in the input. Thus, an algorithm that ranked all start nodes above all other network nodes would have a start node fraction of 1. To quantify tendency to return hub nodes, we calculated the median degree of output nodes where degree was the total number of edges connected to the node.

### Cross-validation and target validation

Ten repeats of 10-fold cross-validation were performed for each data set to calculate the area under the ROC curve (AUC). Each data set was divided into tenths, with one tenth left out each time; then that process was repeated ten times for a total of 100 lists each with 90% of the original input list. Sensitivity and specificity were found using the omitted 10% of nodes as "true" nodes to be found by the algorithms. We also as examined Fraction Recovered as the fraction of left out nodes recovered in the top nodes (top 200 nodes for node prioritization or any node present in a subnet for subnet id algorithms). When omitted input nodes were not included in the network, they were excluded from the list of "true" nodes, as the use of that network prevented them from being included in the output regardless of the algorithm used.

For connectivity map data, sensitivity, specificity, and fraction recovered were calculated based on ranking of known drug targets in algorithm outputs where known drug targets were determined as described previously [25].

### Empirical null distributions

To determine whether nodes were highly ranked based on the network properties only (irrespective of the input list) we generated lists of randomly selected input nodes. Fold changes were chosen from a random distribution with mean 0 and standard deviation 1, with corresponding p-values. Fold change and p-value pairs were randomly assigned to all possible nodes, and the nodes with highest fold change were used as the input list. We generated 10,000 random gene lists each of length 200 and ran the algorithms on these input lists. We were thus able to determine, for each node and algorithm, the frequency each node was ranked higher than a chosen output rank.

## Supporting information

S1 Fig Performance results using standard summary statistics (mean and standard deviation across datasets) for AUROC (left) and Fraction Recovered (right). Comparison of algorithms was difficult due to variation across datasets. Thus, a rank-based approach was used to establish the fraction of datasets for which the algorithm was performing in top five algorithms for each dataset (Fig 2C and 2D).
(EPS)

S2 Fig Fraction of times a node was highly ranked using randomly chosen input start nodes as a function of node degree. Causal regulator algorithms consider each node in two directions–positive (black points) and negate (red points).
(EPS)

S3 Fig Characterization and performance results generalize across the HumanNet published, undirected network. Average fraction of start nodes in the output (A) and median degree (B) characterization of each algorithm. Cross-validation performance of algorithms as indicated by the fraction of datasets for which the algorithm appeared in the top five when ranked by AUROC (C) or Fraction recovered (D) from the CRISPR screen hits, Genetic Association, and KEGG/REACTOME datasets using HumanNet as the network. Note: Because HumanNet contains no signed or directed edges, the causal regulator algorithms were not examined in this analysis.
(EPS)

S1 Table Algorithm parameters used (missing algorithms did not have adjustable parameters). (DOCX)

S1 Data Summary values to create plots in Fig 2 and S1 Fig. (CSV)

S2 Data Summary values to create plots in Fig 3. (CSV)
