# Supplementary material for: Benchmarking network algorithms for contextualizing genes of interest
Source: PLoS Comput Biol. 2019 Dec 20;15(12):e1007403. doi: 10.1371/journal.pcbi.1007403 (PMC6944391; doi:10.1371/journal.pcbi.1007403)
Supplement: S1 Table — (DOCX) [file pcbi.1007403.s004.docx]

**Supplementary Table 1**: algorithm parameters used (missing algorithms did not have adjustable parameters)

| **Algorithm** | **Parameters** |
| --- | --- |
| Network Propagation | alpha = 0.5, L1Threshold=0.000001 |
| Random Walk | r = 0.5, L1Threshold=0.000001 |
| Neighborhood Scoring | alpha = 0.5 |
| ToppNet HITS | r = 0.5, iterations=100, eps=0.0001 |
| ToppNet KM | K = 4 |
| Active Modules | numberOfModules = 10, iterations=10000, activationProbability=0.5, startTemp=1000, dmin=10, backgroundRuns=100 |
| Pathway Inference | beta = 1, d=2, iterations=100, |
| HotNet | iterations = 100, t=0.1, d=0.01, minSize=3, sizeLimit=10000 |
| HotNet2 | iterations = 100, d=0.01, min.size=3, delta.max.size=10 |
| DIAMOnD | n = 200, alpha=1 |
| Causal Reasoning | maxSteps = 2 |
| SigNet | maxSteps = 2 |
